# Sense of Coherence and Physical Health. The Emotional Sense of Coherence (SOC-E) was Found to be the Best-Known Predictor of Physical Health

**DOI:** 10.1100/tsw.2006.344

**Published:** 2006-06-22

**Authors:** Trine Flensborg-Madsen, Søren Ventegodt, Joav Merrick

**Affiliations:** ^1^ Quality of Life Research Center, Teglgårdstræde 4-8, DK-1452 Copenhagen K, Denmark; ^2^ Research Clinic for Holistic Medicine, Beer-Sheva, Israel; ^3^ Nordic School of Holistic Medicine, Copenhagen, Denmark; ^4^ Scandinavian Foundation for Holistic Medicine, Sanvika, Norway; ^5^ Interuniversity College, Graz, Austria; ^6^ National Institute of Child Health and Human Development, Beer-Sheva, Israel; ^7^ Center for Disability and Human Development, Faculty of Health Sciences, Ben Gurion University, Beer-Sheva, Israel; ^8^ Office of the Medical Director, Division for Mental Retardation, Ministry of Social Affairs, Jerusalem, Israel

**Keywords:** Antonovsky, sense of coherence scale, emotionality, mentality, physical health, Denmark

## Abstract

In this study, we created a measure for emotionality named the emotional sense of coherence (SOC-E). We found that SOC-E was significantly associated with physical health (r = 0.266; *p* < 0.05), while it was not significantly associated with psychological health (r = 0.006; NS). Based on a correlation matrix, we constructed a new scale, the SOC-E II, which was even better associated with physical health (r = 0.362) and also associated with psychological health (r = 0.259; *p* < 0.01). Our results showed that SOC-E and SOC-E II were better predictors of physical health than the SOC scales developed by Aaron Antonovsky (1923–1994) (SOC-29 and SOC-13). We conclude that emotional coherence is important for physical health, but it is not important in the same way for psychological health. In a previous study, we found that a mental operationalization of Antonovsky's sense of coherence was highly associated with psychological health and not associated with physical health. Based on these two studies, we conclude that physical health is primarily associated with emotions, while psychological health is primarily associated with mentality.

### INTRODUCTION

In a previous study, we returned to the very original theory of Aaron Antonovsky (1923–1994) from the Faculty of Health Science, Ben Gurion University of the Negev in Beer-Sheva and created a questionnaire based on the theory and the conceptualization as described by him[1]. With this questionnaire, which included questions that were predominantly mental, we tested the hypothesis of Antonovsky and what had been proved in several studies: that there was a connection between sense of coherence (SOC) and physical health. We expected to find quite a strong association between the original questions, combined into a 9-item SOC scale, and physical health. Surprisingly, we found no association whatsoever; the only significant correlation found was between our scale and psychological health[2]. The study made us wonder how we could explain the fact that the mental questions based on the original theory of Antonovsky showed no correlation to physical health. These reflections are the foundation for our theory that differentiates clearly between *mentality* and *emotionality*. We recognize three entities of the human being carrying consciousness: the human wholeness (the "soul"), the brain-mind (the "ego" and the "mental" functions), and the body-mind (the "id"). Some researchers argue that from a psychological perspective, the "emotional" realm of man is part of the more general "mental" domain. From our perspective, the "mental" functions are dominated by brain function (including somatosensoric modalities), while "emotional" functions are related to the body-mind (which also contains the sensations related to sexuality). The concept of "the human psyche" contains all these dimensions, including the dimensions of consciousness related to the human wholeness (or "soul").

Several studies have proven the association between emotional aspects and health using different varieties of emotionality measures; examples are negative affective states[3,4,5,6], positive emotions[7], positive affect[8], optimism[9], affect balance[10], cheerfulness[11], Emotional Expression and Control Scale (EEC)[12], and alexithymia[12]. In this study, we constructed an emotional measure based on our comprehension of emotionality, inspired by the co-founder of the Coaches Training Institute, Henry Kimsey-House[13]. Our measure was based on the feelings of anger, gladness, sorrow, and sexual desire and the intensity, permission, and impact aspects thereof (see Table 1). We named this new measure the Emotional Sense of Coherence (SOC-E) because we consider it to be a measure of how to be emotionally coherent with yourself and the world. SOC-E was tested in a pilot study comprising 113 subjects to investigate whether emotionality contrary to mentality is in fact a strong predictor of physical health.

Our hypothesis in this study was that we would find a significant correlation between SOC-E and physical health. However, we did not expect the correlation to be very strong between SOC-E and psychological health.

### METHODS

Structured questionnaires were distributed to randomly selected persons at three different locations (respondents aged 20 years or older): at the entrance hall of the University Medical Center (Rigshospitalet) in Copenhagen (57 replies, including patients, visitors, nurses, physicians, and other staff); at the Faculty of Medicine "Panum Institute" in Copenhagen (40 replies); and at the Copenhagen Business School (23 replies). The questionnaires were handed out and about 90% of the people who were asked to fill out the questionnaire agreed. Of the 120 returned questionnaires, 113 were filled out; the remaining 7 were half-filled or not filled out. The respondents returned the questionnaires in a full-size envelope after time alone to fill out the questionnaire.

### Measurements

- **Self-reported physical health:** Measured by the following question: *"How do you consider your physical health to be at the moment?"* — (1) very good; (2) good; (3) neither good nor bad; (4) bad; (5) very bad.
- **Self-reported psychological health:** Measured by the following question: *"How do you consider your psychological health to be at the moment?"* — (1) very good; (2) good; (3) neither good nor bad; (4) bad; (5) very bad. (Thus, both health measures are inverse scales.)
- **Gender**: Measured as: (1) male; (2) female.

**TABLE 1**
**Construction of the SOC-E Scale**

| | Intensity | Permission | Impact |
|---|---|---|---|
| Anger (inside) | When you get angry, to what degree do you feel that anger? | To what degree do you give yourself the permission to feel anger? | To what degree does your anger affect you? |
| Anger (outside) | | To what degree do you give yourself the permission to express anger? | To what degree does your anger affect people? |
| Gladness (inside) | When you get happy, to what degree do you feel that gladness?* | To what degree do you give yourself the permission to feel gladness?* | To what degree does your gladness affect you? |
| Gladness (outside) | | To what degree do you give yourself the permission to express gladness?* | To what degree does your gladness affect people? |
| Sorrow (inside) | When you feel sad, to what degree do you feel that sorrow? | To what degree do you give yourself the permission to feel sorrow? | To what degree does your sorrow affect you? |
| Sorrow (outside) | | To what degree do you give yourself the permission to express sorrow? | To what degree does your sorrow affect people? |
| Sexual desire (inside) | When you feel sexual desire, to what degree do you feel that desire?* | To what degree do you give yourself the permission to feel sexual desire?* | To what degree does your sexual desire affect you? |
| Sexual desire (outside) | | To what degree do you give yourself the permission to express sexual desire?* | To what degree does your sexual desire affect people? |

* The six items to be used in SOC-E II (see Table 8).

- **Age**: Divided into the groups: (1) 20–30 years; (2) 31–40 years; (3) 41–50 years; (4) 51–60 years; (5) 61–70 years; (6) >70 years.
- **Emotional sense of coherence (SOC-E):** Respondents were presented with a list of 20 questions in the categories of *anger, gladness, sorrow,* and *sexual desire* with five questions in each category. The idea behind these questions is presented in Table 1. The questions were asked on a five-point scale with the options of replying: (1) very high degree, (2) high degree, (3) neither high nor low, (4) low degree, (5) very low degree.

In order to build a total SOC-E score for each respondent, an inverse SOC-E scale was constructed where it was possible to score between 20 and 100 points, with 20 points implying that one perceived feelings as being high in intensity, permission, and impact, and 100 points implying that the SOC-E was very low. The internal reliability was tested in relation to interitem reliability, which showed that the majority of the 20 items were positively correlated. The only items that were not positively correlated with the other items were some of the questions concerning sexual desire, but these negative correlations were very small and insignificant. The Cronbach Alpha coefficient was found to be 0.847, which points to a high degree of parallelism in the subjects' response patterns, but not so high that one should discard items that appear well founded on a priori grounds. The distribution of respondents according to the scale, SOC-E, was approximately normal (see Fig. 1). The mean score was 44.30 points and the standard deviation was 9.42.

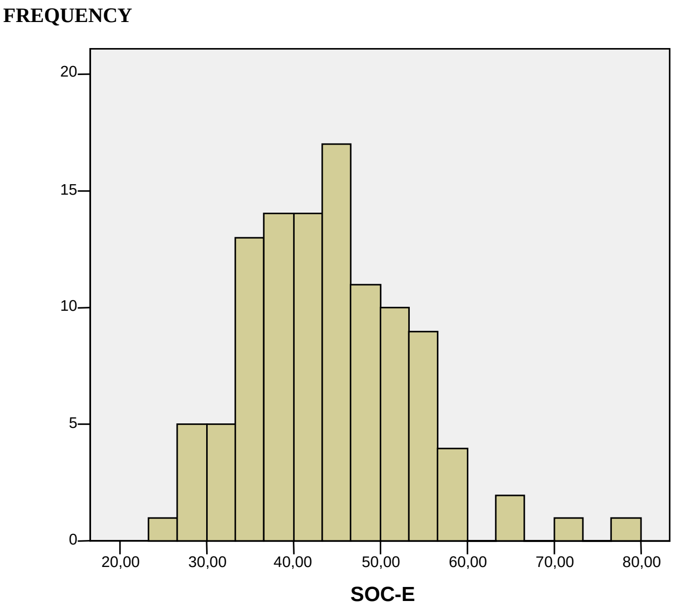

**FIGURE 1.** The distribution of respondents according to SOC-E.

### Statistical Methods

Even though significant differences were found in the demographic characteristics of the responders at the three different locations (see Table 2), the data were analyzed as a whole because of the relatively low number of respondents.

**TABLE 2**
**Means for Dependent and Independent Variables at Different Locations.**

| | Medical School (n = 35) | Business School (n = 23) | Entrance Hall, Hospital (n = 55) |
|---|---|---|---|
| Physical health (1 = best, 5 = poorest) | 1.6 | 2.3 | 2.3 |
| Psychological health (1 = best, 5 = poorest) | 1.7 | 2.5 | 2.6 |
| SOC-E (20 = best, 100 = poorest) | 42.4 | 45.0 | 45.2 |
| Sex (1 = male/2 = female) | 1.8 | 1.6 | 1.5 |
| Age, years | 22.6 | 33.0 | 45.9 |

In the first stage, bivariate analyses were performed between the dependent variables physical health and psychological health and the independent variables SOC-E, and the subgroups anger, gladness, sorrow, and sexual desire and also intensity, permission, and impact (see Table 3). Note that the former four and the latter three subscores are based on the same replies from each participant, only summed horizontally vs. vertically according to Table 1. In addition, correlations were found between SOC-E and the variables gender and age (see Table 4). In the bivariate analyses, Pearson correlation was used. In the second stage, linear regression analyses were performed with each of the two health measures as dependent variables, and with SOC-E, age, and gender as independent variables (see Tables 5 and 6).

**TABLE 3**
**Pearson Correlation Coefficients Between SOC-E and Health Measures**

| | Physical Health | Psychological Health |
|---|---|---|
| **SOC-E (total score)** | **0.266*** | **0.006 (NS)** |
| Intensity | 0.324** | 0.052 (NS) |
| Permission | 0.205* | 0.061 (NS) |
| Impact | 0.182 (NS) | –0.085 (NS) |
| Anger | 0.008 (NS) | –0.101 (NS) |
| Happiness | 0.301* | 0.221* |
| Sorrow | 0.078 (NS) | –0.256* |
| Sexual desire | 0.289* | 0.203* |

* $p < 0.05$.
** $p < 0.001$.

**TABLE 4**
**Pearson Correlation Coefficients Between SOC-E, Gender, and Age**

| | SOC-E |
|---|---|
| Sex | –0.349* |
| Age | 0.188 (NS) |

* $p < 0.01$; Male SOC-E mean = 48.95 (SD = 9.56); Female SOC-E mean = 42.09 (SD = 8.51).

**TABLE 5**
**Linear Regression with Physical Health as Dependent Variable**

| | B | S.E. | Sig |
|---|---|---|---|
| SOC-E | 0.024 | 0.011 | 0.03 |
| Age (10-years interval) | 0.155 | 0.068 | 0.03 |
| Gender | –0.099 | 0.222 | 0.66 |

**TABLE 6**
**Linear Regression with Psychological Health as Dependent Variable**

| | B | S.E. | Sig |
|---|---|---|---|
| SOC-E | 0.001 | 0.011 | 0.94 |
| Age (10-years interval) | 0.098 | 0.071 | 0.17 |
| Gender | 0.097 | 0.233 | 0.68 |

In order to illustrate which factors seemed to be most associated with physical health, we made a correlation matrix, Table 7, on which we tentatively created a new measure, the SOC-E II, in order to create a measure that described physical health in the best possible way. All analyses were carried out using the statistics program SPSS version 12.

**TABLE 7**
**Correlation Matrix, Illuminating which Parts of SOC-E Have the Greatest Association with Physical Health**

| | | Physical Health | Intensity | Gladness | Sexual Desire | Permission | Impact | Sorrow | Anger |
|---|---|---|---|---|---|---|---|---|---|
| **Physical Health** | Pearson Cor. | 1 | **0.32** | **0.30** | **0.29** | **0.21** | 0.18 | 0.08 | 0.01 |
| | *p*-value | | **<0.0005** | **<0.005** | **<0.005** | **<0.05** | 0.06 | 0.42 | 0.94 |
| **Intensity** | Pearson Cor. | **0.32** | 1 | 0.51 | 0.56 | 0.63 | 0.57 | 0.45 | 0.55 |
| | *p*-value | **<0.0005** | | <0.0005 | <0.0005 | <0.0005 | <0.0005 | <0.0005 | <0.0005 |
| **Gladness** | Pearson Cor. | **0.30** | 0.51 | 1 | 0.30 | 0.57 | 0.61 | 0.32 | 0.24 |
| | *p*-value | **<0.005** | <0.0005 | | <0.005 | <0.0005 | <0.0005 | <0.005 | <0.005 |
| **Sexual Desire** | Pearson Cor. | **0.29** | 0.56 | 0.30 | 1 | 0.53 | 0.50 | 0.08 | 0.11 |
| | *p*-value | **<0.005** | <0.0005 | <0.005 | | <0.0005 | <0.0005 | 0.38 | 0.,27 |
| **Permission** | Pearson Cor. | **0.21** | 0.63 | 0.57 | 0.53 | 1 | 0.65 | 0.64 | 0.62 |
| | *p*-value | **<0.05** | <0.0005 | <0.0005 | <0.0005 | | <0.0005 | <0.0005 | <0.0005 |
| **Impact** | Pearson Cor. | 0.18 | 0.57 | 0,61 | 0.50 | 0.65 | 1 | 0.68 | 0.56 |
| | *p*-value | 0.06 | <0.0005 | <0.0005 | <0.0005 | <0.0005 | | <0.0005 | <0.0005 |
| **Sorrow** | Pearson Cor. | 0.08 | 0.45 | 0.32 | 0.08 | 0.64 | 0.68 | 1 | 0.42 |
| | *p*-value | 0.42 | <0.0005 | <0.0005 | 0.38 | <0.0005 | <0.0005 | | <0.0005 |
| **Anger** | Pearson Cor. | 0.01 | 0.55 | 0.24 | 0.11 | 0.62 | 0.56 | 0.42 | 1 |
| | *p*-value | 0.94 | <0.0005 | 0.01 | 0.27 | <0.0005 | <0.0005 | <0.0005 | |

### RESULTS

The SOC-E scale is defined to range from 20–100 points; the mean score in this study was 44.30 point (SD = 9.42). The mean SOC-E scores together with means from the other variables are shown in Table 2 for the three different locations at which the questionnaires were distributed.

Table 2 shows that the respondents from the medical school generally have better health than the other respondents. Furthermore, the group has a lower SOC-E score, contains more females, and is younger.

Table 3 presents the results of the bivariate analysis in which we looked at the Pearson correlation coefficients between the variables. Note that a positive correlation signifies that lack of SOC-E goes with lack of health, i.e., the hypothesized direction of the association. Table 4 shows the correlation coefficients between SOC-E and gender and age.

Table 3 shows that physical health is strongly correlated with SOC-E, while there is no significant correlation between SOC-E and psychological health. The two health measures showed dissimilar patterns according to the subgroups. Physical health was significantly correlated with the intensity ($r = 0.324$; $p < 0.001$) and permission ($r = 0.205$; $p < 0.05$) of feelings, but less (and not significant) with the impact, while psychological health was not correlated with any of these measures. Physical health was significantly correlated with happiness (0.301; $p < 0.001$) and sexual desire (0.289; $p < 0.05$), but not with anger and sorrow. Psychological health was correlated positively to the feelings of happiness and sexual desire, and negatively correlated to sorrow. Table 4 shows that SOC-E was significantly associated with gender so that men generally have a higher SOC-E score, meaning a low degree of intensity, permission, and impact of their emotions.

Table 5 shows a linear regression analysis with physical health as the dependent variable, and age and gender as the independent variables. SOC-E was significantly associated with physical health even after stratifying for age and gender; the B estimate implied that a 2*SD change in SOC-E was followed by a 0.45 units change in physical health on the 1–5 scale (as 2*9.42*0.024 = 0.45). As could be expected, age seemed to be associated with physical health so that older people have poorer health. Table 6 shows a similar analysis, only with psychological health as the dependent variable. The variables SOC-E, age, and gender all turned out to be insignificant in the analysis, which confirms the results from the bivariate analysis, where there was an insignificant correlation between SOC-E and psychological health.

In order to illustrate which subparts of the SOC-E scale have the greatest impact on physical health and to demonstrate the interitem correlations, we constructed a correlation matrix that included the four feelings: anger, gladness, sorrow, and sexual desire in addition to the intensity, impact, and permission of these, see Table 7.

Table 7 illustrates that the areas that are most important to physical health are the feelings of happiness and sexual desire in addition to the intensity and permission of one’s feelings. However, the feelings of anger and sorrow do not seem to be associated with physical health and neither does the impact of one’s feelings. To test whether we could find a measure that was better associated with health than SOC-E, we made a scale that included the six questions (marked in Table 1): intensity of gladness and sexual desire; and the “inside” and “outside” aspects of the permission of gladness and sexual desire — we call it the SOC-E II. The Cronbach’s Alpha for SOC-E II was 0.788, which is rather high since the scale only contains six items. The scale is defined to range from 6–30 points, the mean is 11.76 (SD = 3.49). The results from the bivariate analyses are shown in Table 8.

**TABLE 8**
**Pearson Correlation Coefficients Between SOC-E II and Health**

| | Physical Health | Psychological Health |
|---|---|---|
| SOC-E II | 0.362* | 0.259** |

* $p < 0.01$ by construction.
** $p < 0.01$.

## DISCUSSION

### Method Issues

The cross-sectional design is the main shortcoming of the study. In addition, the small study population was not representative for the general population as the questionnaires were distributed at two education sites and a medical center. It was possible to adjust for the variables of gender and age, but there was no available information about other variables that could have had an influence on the association between SOC-E and health. The Cronbach’s Alpha coefficient for SOC-E was found to be 0.847, pointing towards a high degree of parallelism in the subjects’ response patterns, and Cronbach’s Alpha for SOC-E II was 0.788, which is rather high as the scale contains only six items.

### Result Issues

This paper is the last one in a series of six papers describing the association between sense of coherence (SOC) and physical health. A review paper we found showed that most studies have only found correlations of 0.2–0.3 between SOC and physical health, and several studies found the association to be insignificant[14]. By eliminating the notion of predictability and creating a new SOC scale, we were able to find a correlation of 0.282 ($p < 0.0005$) between SOC and physical health and a correlation of 0.578 ($p < 0.0005$) between SOC and psychological health[15]. In a pilot study including 100 respondents, we tested the original theory of Antonovsky by creating a 9-item SOC scale based on his original theory[2]. We found a correlation of only 0.044 (NS) to physical health and a correlation of 0.502 ($p < 0.0001$) to psychological health, and since our 9-item SOC scale was thoroughly mental in its operationalization, the study made us conclude that mentality is associated with psychological health, while we believed emotionality to be associated with physical health. This could also explain the fact that various studies have not been able to find any associations between the 29-item SOC scale and physical health, since we believed that the original scale mixed up mental and emotional aspects[16]. In this paper, we investigated whether we could actually verify our hypothesis of a strong association between emotionality, SOC-E, and physical health.

We constructed a measure for emotionality, the emotional sense of coherence (SOC-E), that included the four feelings of anger, gladness, sorrow, and sexual desire, and the three dimensions of intensity, permission, and impact. Our hypothesis in this study was that SOC-E was strongly associated with physical health, while we believed that it was not as strongly associated with psychological health. Our study confirmed our hypothesis, since we showed that SOC-E was significantly associated with physical health ($r = 0.266$; $p < 0.05$) and that it was not associated with psychological health ($r = 0.006$; NS). The correlation between SOC-E and physical health was moderate, however, it was higher than the majority of studies that have investigated the association between Antonovsky’s definition of sense of coherence (SOC-29 and SOC-13) and physical health have found[14,17,18,19,20,21,22,23,24]. We therefore consider our result to be worthy of note.

Using linear regression analysis, we showed that the association between SOC-E and physical health stayed significant even after controlling for age and gender. According to psychological health, the regression analysis confirmed our hypothesis and our results from the simple correlation coefficient: that SOC-E was not associated with psychological health. Based on the correlation matrix, but without any multivariate “data massage”, we constructed the measure SOC-E II that included the intensity and permission of the feelings gladness and sexual desire. We found that SOC-E II was significantly associated with both physical health ($r = 0.362$; $p < 0.01$) as well as psychological health ($r = 0.259$; $p < 0.01$). Hence, we succeeded in finding a measure of six questions, SOC-E II, that was highly associated with physical health — the coefficient was higher than the majority of studies have found between Antonovsky’s 29-item SOC scale and physical health[14,17,18,19,20,21,22,23,24].

Several other studies have investigated the association between emotions and health[3,4,5,6,7,8,9,10,11,12]. Among the emotional measures that have been used is the emotional expression and control scale (EEC) that in many ways resembles the measures SOC-E and SOC-E II that we used in the present study. EEC is an 18-item scale developed by Bleiker et al.[25] that yields subscale scores for emotional expression-in, emotional expression-out, and emotional control. The subscale of emotional control has shown to be positively correlated with bowel symptoms[12], so that patients with a tendency to actively control their emotions subjectively experienced fewer bowel and systemic symptoms, and better emotional functioning. In addition, the patients who were most aware of their subjective feelings, but also exerted the greatest control over their emotional reactions, enjoyed a higher quality of life[12].

In this study, we found that the emotions of gladness and sexual desire were associated with physical health, and similar results have been found in other studies. Positive emotions have been found to be associated with survival rates[8], increased survival[9], and longevity[7], while symptoms of depression were found to predict decreased survival in coronary artery disease patients[6,26]. We found that the intensity and permission of feelings were associated with physical health, which is also in accordance with other findings. A study suggested that when individuals must actively inhibit emotional expression, they are at increased risk for a variety of health problems[27]. Difficulty recognizing and expressing emotions was found to predict an increase of perceived disease activity[28], and difficulty in identifying and describing emotions was predictive of a decrease in physical functioning, all-cause mortality, and poor treatment outcome in cancer patients[29]. Experimental studies have shown that health outcome improved after a decrease in alexithymia[30] and after emotional expression was encouraged[31], and it has been found that the way in which patients with rheumatoid arthritis regulate their emotions was associated with the way they perceived their health[28]. A weak association has been found in a longitudinal study between a high score on an antiemotionality scale and the development of breast cancer[32], and a study investigating risk factors of cancer suggests that antiemotionality may be an important distinctive personality characteristic in patients with cancer[33]. Expressing emotions has also proven to be associated with fewer medical appointments, enhanced physical health, and vitality for women with breast cancer[29].

We conclude from this study that SOC-E and, tentatively, SOC-E II are reliable emotionality measures that both seem to have great associations with physical health. However, a prospective study is needed to determine the causality, as it would be preferable to incorporate a bigger population and to integrate further possible confounders. Physical and psychological health are often considered to be closely interrelated, or even put together in the same health measure as for example SCL-90[34,35] or SIRS[36]. However, we believe that it is essential to separate the two health measures, as we also believe that it is essential to separate mentality and emotionality in a measure of SOC. Undoubtedly, physical health and psychological health are interrelated to some degree and so is mentality and emotionality. The conclusions of the present paper, as well as our previous paper[2] are, therefore, simplified as we conclude emotionality to be associated with physical health and mentality to be associated with psychological health. However, we believe that the significant reverse results we have found in the two papers are noteworthy because of their obvious symmetry.

### CONCLUSION

In this study, we created the emotionality measure, the emotional sense of coherence (SOC-E), which included the four feelings of anger, gladness, sorrow, and sexual desire and the three dimensions of intensity, permission, and impact. We found that SOC-E was significantly associated with physical health and that it was not associated with psychological health, which was in accordance with our hypothesis. Based on a correlation matrix, we constructed the SOC-E II, which was even better associated with physical health than SOC-E and also associated with psychological health.

Our findings are in agreement with several other studies concluding that emotions are significantly associated with health. In a previous study investigating the original theory of Antonovsky, we found that a mental operationalization of SOC was highly associated with psychological health, but not associated with physical health; hence, the results were reverse in the two studies. Based on both studies, we conclude that physical and psychological health must have different etiologies: physical health has to do with emotions, while psychological health has to do with mentality.

### BIOSKETCHES

**Trine Flensborg-Madsen**, **BSc, MSc** (Public Health Science), is currently a research assistant at the Institute of Preventive Medicine, Faculty of Health Sciences, University of Copenhagen, where she is finishing her PhD. Earlier a research assistant at the Quality of Life Research Center in Copenhagen, where she participated in several research projects concerned with holistic health, sense of coherence, and research and follow-up of the Copenhagen Birth Cohort 1959–61. E-mail: tf@imp.hosp.dk Website: http://www.ipm.hosp.dk/person/tfm.htm

**Søren Ventegodt, MD,** is the director of the Nordic School of Holistic Health and Quality of Life Research Center in Copenhagen, Denmark. He is also responsible for a Clinical Research Clinic for Holistic Medicine in Copenhagen and is a popular speaker throughout Scandinavia. He has published numerous scientific or popular articles and a number of books on holistic medicine, quality of life, and quality of working life. His most important scientific contributions are the comprehensive SEQOL questionnaire, the very short QOL5 questionnaire, the integrated QOL theory, the holistic process theory, the life mission theory, and the ongoing Danish Quality of Life Research Survey, 1991–94 in cooperation with the University Hospital of Copenhagen and the late professor of pediatrics, Bengt Zachau-Christiansen, MD, PhD. E-mail: ventegodt@livskvalitet.org. Website: www.livskvalitet.org

**Joav Merrick, MD, MMedSci, DMSc,** is professor of child health and human development affiliated with the Center for Multidisciplinary Research in Aging, Zusman Child Development Center, Division of Pediatrics and Community Health at the Ben Gurion University, Beer-Sheva, Israel; the medical director of the Division for Mental Retardation, Ministry of Social Affairs, Jerusalem; and the founder and director of the National Institute of Child Health and Human Development. Dr. Merrick has numerous publications in the field of child health and human development, rehabilitation, intellectual disability, disability, health, welfare, abuse, advocacy, quality of life, and prevention and received the Peter Sabroe Child Award for outstanding work on behalf of Danish Children in 1985 and the International LEGO-Prize (“The Children’s Nobel Prize”) for an extraordinary contribution towards improvement in child welfare and well being in 1987. E-mail: jmerrick@internet-zahav.net. Website: www.nichd-israel.com
